# Association between Physical Activity and Fundamental Movement Skills in Preschool-Aged Children: Does Perceived Movement Skill Competence Mediate This Relationship?

**DOI:** 10.3390/ijerph18031289

**Published:** 2021-02-01

**Authors:** Qing He, Johan Y. Y. Ng, John Cairney, Chloe Bedard, Amy S. C. Ha

**Affiliations:** 1 Faculty of Education, The Chinese University of Hong Kong, Hong Kong 999077, China; qinghe@cuhk.edu.hk (Q.H.); yyng@cuhk.edu.hk (J.Y.Y.N.); 2 School of Human Movement and Nutrition Sciences, University of Queesland, Brisbane 4072, Australia; j.cairney@uq.edu.au; 3 School of Public health and Health Systems, University of Waterloo, Waterloo, ON N2L 3G1, Canada; c3bedard@uwaterloo.ca; 4 Department of Sports Science and Physical Education, Faculty of Education, The Chinese University of Hong Kong, Hong Kong 999077, China

**Keywords:** physical activity, fundamental movement skills, perceived movement skill competence, accelerometry, early childhood

## Abstract

Objectives: The purpose of this research is to examine whether perceived fundamental movement skills (FMS) competence mediated the relationship between actual FMS and physical activity (PA) in Hong Kong preschool-aged children. Design: A cross−sectional study. Methods: 148 preschool-aged children (43% girls; mean age = 4.52 ± 0.67 years) from five preschools/childcare centres completed all assessments. Actual FMS was rated using the Test of Gross Motor Development−2, whilst perceived FMS was assessed via the Pictorial Scale for Perceived Movement Skill Competence. PA was measured through accelerometry. A bootstrap method was used to assess the potential mediating effect of perceived movement skill competence on the relationship between actual FMS and PA. All mediation models were adjusted for sex and age. Results: Mediation analyses showed that the direct path between actual FMS and moderate-to-vigorous physical activity (MVPA) was significant (b = 0.228, *p* = 0.008), as was the path between MVPA and actual FMS (b = 0.214, *p* = 0.008). However, perceived FMS competence did not mediate the association between actual FMS and MVPA in the models. Conclusions: Our results showed evidence of reciprocal pathways between actual FMS and MVPA, reinforcing the need to simultaneously target both domains as part of broader developmental strategies, initiated in early childhood. Unlike emergent adolescence, perceptions of movement skill competence do not play a significant role in influencing the relationship between actual FMS proficiency and MVPA in this developmental period.

## 1. Introduction

Early childhood is a crucial developmental stage for the establishment of a physically active lifestyle [1]. A recent systematic review synthesised evidence from 96 studies on the health implications of physical activity (PA) and indicated that PA participation had been consistently and favourably associated with multiple health indicators in early childhood years [2]. The development of consistent healthy habits during childhood increases the possibility of carrying over such patterns into adulthood [3]. Although evidence has manifested the importance of PA participation during early childhood, some previous surveys have revealed that pre-schoolers do not sufficiently engage in PA [2,4]. Accordingly, in order to understand the low levels of PA in this critical developmental period, we must first identify salient correlates of the behaviours, which is crucial for the development strategies for improving the health of young children.

The development of fundamental movement skills (FMS) is vital to children’s healthy weight and engagement in PA [5]. Increasing evidence has strongly supported this idea [6,7]. FMS are considered the essential ‘building blocks’ of advanced and sophisticated movement sequences required for a variety of sports, games or physical activities [8]. Early childhood is a critical time for equipping young children with these skills, as movement patterns are just being developed [9]. Stodden and colleagues [5] proposed a development model in which PA in early childhood initially promotes the development of FMS as a basic motor pattern. Further, perceived FMS, an individuals’ perception of their own movement ability, may serve as a mediator between actual FMS proficiency and PA behaviours in this conceptual model [5]. However, the relative contribution of perceived FMS to PA may not be the same across different developmental stages [10]. As such, there is a dearth of empirical evidence to examine the role of perceived FMS in the relationship between actual FMS and PA among children in the early years.

Increasing research suggests that perceived FMS may serve as a mediator between actual FMS competence and PA. Barnett and colleagues [11] found that object control skill proficiency was mediated through perceived sports competence to influence PA level in adolescence. Perceived movement skill competence has also been shown to be a mediator in the relationship between actual FMS competence and self-reported PA in Hong Kong Chinese primary school children [12]. Whether perceived FMS mediates the association between actual FMS proficiency and PA engagement in early childhood has not yet been thoroughly investigated. In fact, limited studies in preschool-aged children could be located, and the results suggested that perceived physical competence did not play a mediating role in the relationship between actual FMS and moderate-to-vigorous physical activity (MVPA) [13]. However, the perception of competence tool used did not specifically address the object control skills in the TGMD-2. Thus, whether perceived FMS plays a mediating role in the relationship between actual FMS and PA behaviours remains unclear.

In addition to assessing whether perceived FMS mediates the association between actual FMS and PA in early childhood, it is also imperative to consider that the pathways connecting actual FMS and PA may indeed be reciprocal [6]. Barnett and colleagues [14] found a reciprocal relationship between object control skill competence and MVPA among adolescents around the age of 16. Moreover, Crane and colleagues [13] also found a bidirectional relationship between object control skill competence and MVPA among Canadian pre-schoolers. Given the relationship between actual FMS proficiency and PA is likely to be reciprocal; therefore, this research explored bidirectional pathways between these constructs.

To date, few accelerometer-based PA data have been published for preschool-aged children in the Hong Kong Chinese environment. We, therefore, have little evidence if Hong Kong pre-schoolers meet the current World Health Organization (WHO) PA guideline. Further, limited studies have examined the associations among perceived and actual FMS competence and PA behaviours in this population. Given the gaps in current literature, this research aimed to: (1) Examine whether perceived FMS mediates the relationship between actual FMS proficiency and MVPA levels; and (2) probe the directional associations between actual FMS proficiency and MVPA participation among Hong Kong pre-schoolers. Bridging this gap may proffer insights for further health promotion interventions.

## 2. Materials and Methods

### 2.1. Participants

Prior to the commencement of the study, this cross-sectional research was approved by the University and Clinical Research Ethics Committees (Ref: 2018.332). A total of five pre-schools/childcare centres from 4 (i.e., Central and Western, North, Sha Tin and Eastern) of 18 Hong Kong districts participated in the research. Three- to five-year-old children who did not have physical disabilities and were able to participate in regular PA and perform the FMS were included in the recruitment. Informed written parent consent was obtained for 153 preschool-aged children (42% girls; mean age = 4.52 ± 0.67; 59% consent response rate). Data were collected from December 2018 to March 2019.

### 2.2. Sample Size Calculation

A priori power calculation was conducted to estimate the required sample size and the number of pre-schools based on the research questions. Calculations were conducted using G Power 3.1.9.4, with an alpha level set to 0.05, effect size at 0.15 and power at 95%. A 20% drop-out rate was accounted, and therefore a total of 149 pre-schoolers were expected to recruit.

### 2.3. Measures

A total of 153 preschool-aged children wore Actigraph GT3X+ (Actigraph, Pensacola, FL) accelerometers for five consecutive days (including two weekend days), following standardised accelerometer protocols, to assess the time they spent engaging in PA. Given the age group and sporadic and intermittent nature of young children’s PA, the accelerometer measured 15-s epochs, with a sampling frequency of 30 Hz [15]. Cut-points derived from a previous study of the pre-schoolers (MPA: ≥420 counts/15 s, VPA: ≥842 counts/15 s) was used to categorise children’s PAs into moderate-to-vigorous intensity and the time (in minutes) spent engaged in this activity intensity [16]. Children were included in the final data analysis if their valid wear time was not less than three days, including one weekend day and two weekdays, with a minimum wear time of six hours per day [17]. Non-wear time was indicated by 20 min of consecutive zeros [15]. The parents were reminded daily via WhatsApp messages to ensure that their children removed the devices during water-related activities. Of the 153 children, 148 (96.7%) met the wear-time criteria.

Two locomotor skills (i.e., running and horizontal jumping) and two object control skills (i.e., kicking and overhand throwing) were assessed using the second edition of the Test of Gross Motor Development (TGMD-2) [18]. The TGMD−2 is a validated standardised test, which is a process-orientated assessment of 12 items, including a subset of locomotor and object control skills [18]. The TGMD−2 showed an established validity and reliability in preschool-aged children [9]. The four FMS skills were selected because the kindergarten education curriculum guide of Hong Kong focuses on the mastery of these FMS competencies in young children as part of their learning objectives and expectations. Besides, these skills are considered to be the basis of common sports in Hong Kong, such as athletics, ball games [19].

Prior to data collection, a senior trainer on the research team provided a one-day training session for the two FMS examiners on test procedures, techniques and previous experiences in conducting the TGMD−2 protocol. Inter-rater reliability for each skill using the intra-class correlation coefficient (ICC) was assessed following completion of training. The testing took place in each pre-school or childcare centre. The children were given a practice trial before their tests were formally scored. Children conducted two rounds of trials for each skill, and their FMS were marked as 1 (performed correctly) or 0 (not performed correctly) for each component of the skill. Scores of the two rounds of trials were summed to obtain a raw score for each skill, and then the four skill scores for each child were summed to get an actual FMS score. Specifically, the total score for actual FMS (range 0–32) includes the locomotor score (range 0–16) and the object control score (range 0–16) adhering to the TGMD-2 test administration guidelines [18]. The ICCs across the four skills ranged from 0.91 (for kick) to 0.95 (for run). A total of 151 (98.7%) children completed all four FMS tests (two children were absent from the test).

The Pictorial Scale for Perceived Movement Skill Competence (PMSC) [20] was employed to assess the children’s perceived FMS competence. The instrument is explicitly based on the skills tested by the TGMD−2, which builds on previous measures of perceived motor competence [21]. The same four movement skills (i.e., run, horizontal jump, kick, and overhand throw) were measured in the perceived FMS testing. The PMSC showed two illustrations for each skill, specifically, a child performing the movement skill properly and another showing a child performing the skill poorly. At the beginning of the test, for each skill, the examiner first asked the children what movement skill the child in the picture was doing to check if the children recognised the skills represented. If the children did not understand the skill represented in the picture, they were informed as to what the skill was. After making sure children had a good understanding of the skills, then the children were asked to select a picture from the two illustrations that best represented them. Based on the selected picture, the children were further asked to indicate how similar they were to their chosen picture. Their responses were converted into corresponding scores, that is, ‘very good’ (a score of 4), ‘pretty good’ (a score of 3), ‘sort of good’ (a score of 2) and ‘not that good’ (a score of 1). The scores of the four skills were summed up into a perceived FMS score (ranging from 4 to 16). A high score indicated a child with a high perceived FMS competence. This instrument has shown acceptable face validity and test-retest reliability among young Chinese children aged 4–9 years old [22].

### 2.4. Data Analysis

Descriptive analyses are presented as mean ± standard deviation (mean ± SD). Independent *t*-tests were conducted to identify sex differences in actual FMS, perceived FMS, MVPA and light-to-moderate-to-vigorous physical activity (LMVPA). A Pearson bivariate correlation was executed to examine the associations between all the variables. A bootstrapping approach [23] was utilised to investigate the hypothesised mediator variable (i.e., perceived FMS) in the relationship between actual FMS and MVPA (i.e., the predictor variable and outcome variable). Bootstrapping is strongly recommended in recent years for mediation testing because it does not need to satisfy the normality assumption in regression testing, and can be used effectively with small sample sizes [23]. The bootstrapping method provides point estimates and confidence intervals through which the significance or non-significance of mediating effects can be assessed. The point estimate reveals the mean over the number of bootstrap samples, and if zero does not fall within the confidence intervals of the results, we can conclude that there is a significant mediating effect. SPSS version 23 was used for all analyses, and *p* < 0.05 was set as the significance value.

## 3. Results

A total of 148 young children (mean age = 4.52 ± 0.67) had valid PA data and completed all the FMS assessments. A summary of the children’s descriptive statistics is presented in Table 1. The boys (*n* = 85, 57%) slightly outnumbered the girls. On average, the children spent 144.33 min in LMVPA, of which 67.77 min were spent in MVPA per day. Boys were more active than girls in terms of LMVPA and MVPA (*p* < 0.01 for both). Sex differences were also found in actual FMS, especially for object control skills, where the boys scored higher than the girls (*p* < 0.001). Table 2 shows the bivariate correlations between the variables. Several statistically significant correlations were observed, including positive correlations between actual FMS and time spent engaging in MVPA and LMVPA. Moreover, actual FMS was significantly related to perceived FMS.

**Figure 1:**
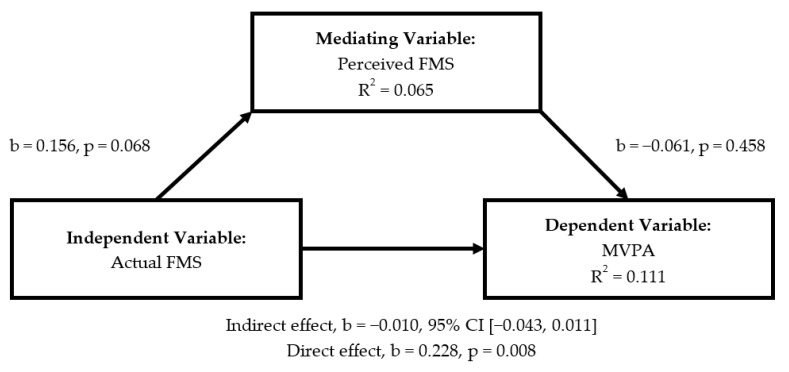
Mediating roles of perceived FMS in the association between actual FMS and MVPA.

**Figure 2:**
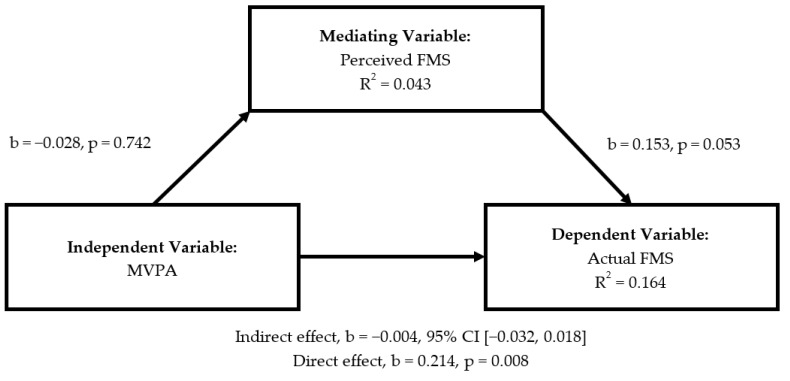
Mediating roles of perceived FMS in the association between MVPA and actual FMS.

There was no significant indirect effect of actual FMS on MVPA through perceived FMS (Figure 1; b = −0.010, 95% CI= [−0.043, 0.011]). However, a significant direct effect existed between MVPA and actual FMS. Specifically, actual FMS competence accounted for 11.1% of the variance in MVPA (b = 0.228, *p* = 0.008). When MVPA served as independent variable (Figure 2), perceived FMS did not mediate association between MVPA and actual FMS (b = −0.004, 95% CI = [−0.032, 0.018]), whilst direct effect revealed that MVPA accounted for 16.4% of the variance in actual FMS (b = 0.214, *p* = 0.008).

## 4. Discussion

In light of the importance of FMS competence and PA participation in early childhood, this current study extends the understanding in this area to investigate the associations among actual FMS, perceived FMS and objectively measured MVPA in the conceptual model [5]. To our knowledge, this is the first study to examine whether perceived FMS mediated the relationship between MVPA and actual FMS in Hong Kong Chinese preschool-aged children. Mixed results were found with regard to our original hypotheses.

One major finding is that perceived FMS did not mediate the relationship between actual FMS and MVPA when age and sex are controlled in this sample. The finding indicated that actual FMS did not indirectly influence the MVPA through children’s perceived FMS, and vice versa. Our results are consistent with the results from a previous study of kindergarten children [13] but differ from the results of studies of children in middle to late childhood [12] and adolescence [14]. The current finding supports the hypothesis of Stodden and colleagues [5], that children in the early years are prone to have unrealistically high perceptions of movement competence because their cognitive abilities are not yet mature enough to accurately evaluate actual FMS competence, unlike the older children and adolescents who are capable of identifying perceived FMS more accurately as their cognitive abilities are more developed. Pre-school children with less social involvement, are less able to formulate their perceptions from the evaluation and feedback of others [24].

There is a relative lack of evidence testing the hypothesised relationship between the perceived FMS and objectively measured MVPA in pre-schoolers [25]. The finding in this study echoes the results of previous research that no relationship between the two variables was found [10,26]. Older children and adolescents with lower FMS may perceive they are not as competent as their peers; as a result, choose not to engage in PA. In contrast, our results suggest that pre-schoolers’ participation in PA is predicted by actual FMS rather than their perceived FMS competence. The absence of mediation effect could be partly explained by the great difficulty to get a reliable measurement of perceived FMS with such young children, who are in the developmental stage of cognition, even if the measurement procedures are performed perfectly. Therefore, compared with perceived FMS, actual FMS are more valuable to drive PA involvement in the early years [5].

Previous studies have not used an aligned assessment tool to explore the association between perceived and actual FMS competence [27,28]. There was no significant association despite the alignment of perceived and actual skill measures in this study. This result contradicts previous research on young children, which found a positive correlation between object control skill and perceived object control skill competence [10,29], but supports the idea that no association exists between actual and perceived FMS competence [5,7]. In this sample, young children tended to overestimate their motor skills (i.e., 50.7% of children scored between 13 and 16 in the total four skills). This lack of variability in the perceived FMS scoring (scoring range 1–4 per skill), resulted in a ceiling effect, which negatively affected our ability to detect a relationship. Given that the present study assessed only four FMS (two locomotor skills and two object control skills), further investigations could measure more skills objectively by matching the assessment tool to determine if perceived FMS serves as a mediator between actual FMS and MVPA.

A secondary aim of the study was to investigate whether there was a reciprocal relationship between actual FMS and MVPA after adjusting for sex and age. This reverse pathway is consistent with a study, Crane and colleagues found the bidirectional relationship between object control skills and PA among children in the kindergarten [13]. The interplay between actual FMS and MVPA in this research contributes new empirical evidence to the conceptual model [5]. In the early years, this reciprocal relationship suggests two inter-related strategies for movement development and health promotion: first, whenever possible, encourage free or unstructured play to promote PA participation. Second, pre-school children should be offered direct or structured play opportunities targeting FMS to encourage greater participation in MVPA. Although our cross-sectional finding cannot decipher cause and effect, the results contribute to our understanding of the potential positive feedback loop between FMS proficiency and MVPA.

Only 12.8% of the pre-schoolers in this study met the criterion based on the new WHO guidelines for PA. Despite the absence of accelerometer-based PA evidence for pre-schoolers in Hong Kong, this result supports a parent-reported survey in Hong Kong, which reported that only 17.7% of preschool-aged children spend one hour per day engaged in outdoor activities [4]. Cultural specificity of Hong Kong pre-schoolers may help explain these findings. Hong Kong is a unique area in China, with a mixed cultural background influenced by Confucian culture, which differs significantly from the Western context. Parents highly regulate their children’s daily activities, and passive obedience is quite common [30]. Parental beliefs (e.g., safety considerations and academic achievement), logistical challenges and even humid climate may have a negative effect on children’s PA engagement [31,32]. As such, it is necessary to examine the influence of parental behaviour, perception and parenting style on young children’s PA behaviours in future studies. Moreover, Hong Kong is a densely populated city with limited space for activities. Participating pre-schools were equipped with limited play areas and lacking professional physical education (PE) teachers to support children to do more free play activities and FMS learning. These may be the factors that contribute to the child’s inadequate PA. Therefore, further research is needed to examine the cultural specificity in PA behaviours and FMS acquisition to design feasible interventions by adjusting measures to local conditions to promote an active lifestyle and FMS development in these populations.

The strengths of this study include: (1) The utilisation of accelerometers to objectively measure PA; (2) the use of a reliable and aligned tool to assess both perceived and actual FMS; (3) and that new information provided support for the conceptual model proposed by Stodden and colleagues [5]. However, several limitations need to be recognised. First, as a cross-sectional study, data were collected at one specific period, so causal relationships between variables could not be examined. Second, a relatively small, non-probabilistic sample of children was recruited in the study; thus, the conclusions may not be generalisable to all the preschool-aged children living in Hong Kong. Third, this research does not control children who participate in some sports discipline or extra-curricular sports activities, which may lead to the actual FMS test results to be inaccurate. Moreover, the current research focuses on a relatively narrow FMS subset in the TGMD−2 and PMSC, which may restrict detecting a significant correlation between perceived and actual FMS, and MVPA. Future research could seek to employ a broader range of FMS test batteries (e.g., combine process and product assessments) to evaluate more comprehensively children’s FMS competence.

## 5. Conclusions

Although perceived FMS does not mediate the association between actual FMS and MVPA, actual FMS directly predicts MVPA behaviour, and vice versa. Children in the early years tend to be inaccurate in terms of their self-perceived FMS owing to immature cognitive processes. Findings in the current research reinforce the conceptual model posited by Stodden et al. that actual rather than perceived movement skill competence was more crucial to MVPA. Longitudinal and experimental designs are required to investigate the causality of these variables further. Pre-school is an ideal time to improve children’s FMS proficiency and PA levels before children develop negative self-perceptions. Follow-up research is needed to target FMS development and increasing PA levels simultaneously in developmental strategies and initiated in early childhood.

## Figures and Tables

**Table 1:** Descriptive statistics for children’s age, physical activity (PA), perceived and actual FMS and sex differences.

Variable	Total (*n* = 148)	Boys (*n* = 85)	Girls (*n* = 63)	P ^a^
	Mean (SD)	Mean (SD)	Mean (SD)	
Age (years)	4.52 (0.67)	4.58 (0.71)	4.43 (0.61)	0.178
Actual FMS (0−32)	16.81 (4.82)	17.56 (4.88)	15.80 (4.57)	0.026
ALM (0−16)	10.21 (2.95)	9.99 (2.77)	10.49 (3.17)	0.321
AOC (0−16)	6.86 (2.93)	7.74 (3.01)	5.68 (2.37)	<0.001
Perceived FMS (4−16)	12.61 (2.64)	12.86 (2.79)	12.29 (2.42)	0.184
PLM (2−8)	6.43 (1.46)	6.62 (1.41)	6.17 (1.49)	0.066
POC (2−8)	6.18 (1.65)	6.24 (1.80)	6.11 (1.42)	0.651
LMVPA (mins)	144.33 (32.79)	151.20 (32.91)	135.07 (30.51)	0.003
MVPA (mins)	67.77 (20.41)	72.19 (21.44)	61.81 (17.37)	0.001

Abbreviation: FMS = fundamental movement skills. ALM = actual locomotor. AOC= actual object control. PLM = perceived locomotor. POC= perceived object control. LMVPA = light-to-moderate-to-vigorous physical activity. MVPA = moderate-to-vigorous physical activity. SD = standard deviation. Note. ^a^ Independent *t*-test examined sex differences.

**Table 2:** Pearson’s bivariate correlations among variables.

Variable	ALM	AOC	Perceived FMS	PLM	POC	MVPA	LMVPA
Actual FMS	0.774 **	0.823 **	0.206 *	0.171 *	0.179 *	0.246 **	0.252 **
ALM		0.420 **	0.223 **	0.165 *	0.213 **	0.139	0.114
AOC			0.172 *	0.168 *	0.127	0.192 *	0.210 *
Perceived FMS				0.831 **	0.870 **	0.019	0.008
PLM					0.449 **	0.028	0.027
POC						0.006	−0.012
MVPA							0.924 **

Abbreviation: FMS = fundamental movement skills. ALM = actual locomotor. AOC = actual object control. PLM = perceived locomotor. POC = perceived object control. LMVPA = light-to-moderate-to-vigorous physical activity. MVPA = moderate-to-vigorous physical activity. Note. * *p* ≤ 0.05, ** *p* ≤ 0.01.
